# The effect of cathodal tDCS on fear extinction: A cross-measures study

**DOI:** 10.1371/journal.pone.0221282

**Published:** 2019-09-18

**Authors:** Ana Ganho-Ávila, Óscar F. Gonçalves, Raquel Guiomar, Paulo Sérgio Boggio, Manish Kumar Asthana, Angelos-Miltiadis Krypotos, Jorge Almeida

**Affiliations:** 1 Proaction Laboratory, Cognitive and Behavior Center for Research and Intervention Faculty of Psychology and Educational Sciences, University of Coimbra, Coimbra, Portugal; 2 Neuropsychophysiology Lab, CiPsi, School of Psychology, University of Minho, Braga, Portugal; 3 Spaulding Neuromodulation Center, Spaulding Rehabilitation Hospital, Harvard Medical School, Boston, MA, United States of America; 4 Social and Cognitive Neuroscience Laboratory and Developmental Disorders Program, Center for Health and Biological Sciences, Mackenzie Presbyterian University, São Paulo, Brazil; 5 Department of Humanities and Social Sciences, Indian Institute of Technology, Roorkee, India; 6 Department of Clinical Psychology, Utrecht University, Utrecht, the Netherlands; BG-Universitatsklinikum Bergmannsheil, Ruhr-Universitat Bochum, GERMANY

## Abstract

**Background:**

Extinction-based procedures are often used to inhibit maladaptive fear responses. However, because extinction procedures show efficacy limitations, transcranial direct current stimulation (tDCS) has been suggested as a promising add-on enhancer.

**Objective:**

In this study, we tested how cathodal tDCS over the right dorsolateral prefrontal cortex affects extinction and tried to unveil the processes at play that boost the effectiveness of extinction procedures and its translational potential to the treatment of anxiety disorders.

**Methods:**

We implemented a fear conditioning paradigm whereby 41 healthy women (mean age = 20.51 ± 5.0) were assigned to either cathodal tDCS (n = 27) or sham tDCS (n = 16). Fear responses were measured with self-reports, autonomic responses, and implicit avoidance tendencies.

**Results:**

Cathodal tDCS shows no statistically significant effect in extinction, according to self-reports, and seems to even negatively affect fear conditioned skin conductance responses. However, one to three months after the tDCS session and extinction, we found a group difference in the action tendencies towards the neutral stimuli (F (1, 41) = 12.04, p = .001, ηp^2^ = .227), with the cathodal tDCS group (as opposed to the sham group) showing a safety learning (a positive bias towards the CS-), with a moderate effect size. This suggests that cathodal tDCS may foster stimuli discrimination, leading to a decreased generalization effect.

**Discussion:**

Cathodal tDCS may have enhanced long-term distinctiveness between threatening cues and perceptively similar neutral cues through a disambiguation process of the value of the neutral stimuli—a therapeutic target in anxiety disorders. Future studies should confirm these results and extend the study of cathodal tDCS effect on short term avoidance tendencies.

## Introduction

According to the classical fear conditioning model of anxiety disorders, anxiety- and fear-related responses are the result of associative learning processes, whereby threatening experiences are associated with originally neutral stimuli that subsequently take on anxiety-inducing properties (fear response acquisition). When individuals are thereafter exposed to these anxiety-inducing stimuli in safe conditions, a new learning occurs, the association between the stimuli and the potential threat is weakened, and the fear response is inhibited or eliminated (fear response extinction).

Current exposure-based treatments for anxiety disorders are still limited in terms of their clinical efficacy with frequent relapse of symptoms [1,2]. In this study, we used non-invasive transcranial direct current stimulation (tDCS) as an add-on intervention to enhance fear-extinction efficacy and observe its impact in three dependent measures that match three distinctive components of the fear response [3]: subjective experience, autonomic responses, and implicit avoidance tendencies.

### Using tDCS to modulate the extinction of fear

One promising way by which fear extinction procedures can be enhanced is through the use of non-invasive brain stimulation techniques. In particular, it has been shown that fear experiences can be modulated by tDCS [4,5]. The modulatory assumptions are that tDCS 1) induces cortical excitability and neuroplasticity, modulating long-term potentiation (LTP) and long-term depression (LTD) mechanisms [6,7]; 2) its effects are polarity specific [8,9] in that anodal stimulation is excitatory, whereas cathodal stimulation is inhibitory when stimulating motor or parietal regions but inconsistent in other brain regions [10]; 3) it interferes with the cortical and subcortical regions involved in fear learning networks and their connectivity patterns [11,12]; and 4) its effects may persist over time [13,14].

Previous literature shows the effect of tDCS in fear responses by modulating the connectivity within the fear network during extinction. For example, the ventro-medial prefrontal cortex (vmPFC) is known to participate in fear learning and amygdala downregulation during fear processing [15] and has been highlighted as a target region in tDCS studies. In particular, it is known that the vmPFC shows increased activity during classical extinction [16,17] and decreased activity during post-reactivation extinction which putatively leads to more or less long-lasting extinction effects [18]. However, because tDCS currents cannot directly access the vmPFC, researchers rely upon the connectivity of the fear network to identify connected and accessible cortical regions [19,20], such as the dorsolateral PFC (DLPFC; [4,19]), or the supra orbital cortex (SOC; [5]).

Studies that stimulate the DLPFC in particular posit that during the acquisition of extinction the DLPFC and the vmPFC are interconnected and simultaneously involved in downregulating fear responses and amygdala activity by means of distinctive functions (emotion regulation and detection value respectively) and through distinctive pathways [5]. Animal models have confirmed and thus showed that whereas the stimulation of the DLPFC leads to increased freezing responses through direct pathways to the intercalated cells in the amygdala [21], the direct stimulation of the vmPFC leads to decreased fear responses through the ITC that in turn have inhibitory projections to the central nucleus of the amygdala [21,22].

Recent studies in humans that observed anodal stimulation of the right DLPFC (rDLPFC) have led to conflicting results. Whereas Abend et al. [23] found anodal tDCS to have no impact in extinction, others found anodal tDCS to enhance both early [19] and late extinction [20]. Moreover, both van’t Wout et al. [20] and Abend et al. [23] found a negative adverse effect of anodal tDCS as it seems to induce generalization of the fear response towards the neutral stimuli.

Similarly, conflicting results emerge when using cathodal tDCS. Although in laboratory cathodal stimulation of the rDLPFC showed no effect in fear extinction in healthy subjects [24], two successful case reports found cathodal tDCS therapeutic effects [25,26], suggesting that cathodal stimulation may be a promising tool in alleviating symptoms of anxiety.

### Is tDCS interfering with the learning of fear extinction or with fear memory reconsolidation?

One possible explanation for the dissonant results comes from the different mechanisms that may be at play: whether extinction occurs inside or outside the reconsolidation time window. Reconsolidation is thought to be the set of processes that occur after memory recall and during which a memory trace is destabilized. According to the reconsolidation theory, when its mechanisms are successfully triggered during its time-window, the memory trace is labile and thought to be susceptible to be strengthened, updated or eliminated [27]. In the case of the elimination of fear-conditioned responses, the PFC structures participate in classical extinction out of the reconsolidation window but are not necessary during reconsolidation [18]. Moreover, PFC participation may even block the mechanisms involved in the long-lasting update of the conditioned fear response [18,28].

Abend et al. [23] suggest that low-frequency (1 Hz) alternating current (AC) stimulation during extinction may have a long-term depression effect on the medial PFC during the reconsolidation window, potentially leading to enhanced extinction. However, and contrary to what expected, the authors found AC stimulation to increase fear response during delayed recall. Similarly, Mungee et al. [24] used cathodal tDCS after fear recall (aimed at triggering reconsolidation), and found no impact on Skin Conductance Responses (SCRs). Of note in Mungee et al [24] study is that participants went through cathodal tDCS immediately after recall (a single presentation of the CS+), and the authors did not employ a fear extinction procedure, during which new information is thought to support the update of the fear memory trace, and to putatively lead to the persistent elimination of the conditioned fear response [29].

### The hypothesis

Following the abovementioned literature, this study aims to verify if cathodal tDCS leads to the extinction of the conditioned fear response, achieved through a reduced participation of the PFC which is putatively associated to the disruption of reconsolidation processes [30,31] or if, on the contrary, cathodal tDCS hinders the extinction of the conditioned fear response [30,31]. If the former, cathodal tDCS over the rDLPFC will lead to the extinction of the different components of the fear response. If the latter, cathodal tDCS will not lead to the extinction of the fear response, both at short- and long-term, and the fear response will be expressed at least by one of the conditioned fear memory indexes (Self-reports, SCRs, AAT. In parallel to the aim of eliminating the fear response components, we aim to observe the effect of cathodal tDCS in the generalization effect towards perceptive or semantic similar stimuli (as unexpectedly happened after anodal tDCS in Abend et al. and Van’t Wout et al studies; [20,23]).

### How to measure fear?

Despite the extensive use of skin conductance responses (SCRs; [4,5]), for measuring fear, relying solely on this data has its drawbacks, one of which is habituation due to repeated presentation of stimuli, resulting in a progressive decrease in SCRs amplitude [28,32]. Habituation to the CSs is a phenomenon that compromises the length of the experimental procedures, the value of the signal across repeated sessions, and the usefulness of the SCRs results [33].

Notwithstanding, previous literature is relatively silent concerning the impact of tDCS in conditioned fear components other than SCRs, with few exceptions on gaze fixation times [34], or neuroimage data [15]. Altogether, the field has overlooked the implicit behavior component of the fear response when examining the efficacy of tDCS on extinction [35,36].

Implicit avoidance tendencies are argued to be action tendencies that are negative atitudes translated into automatic, involuntary, unconscious, goal-independent, and fast affective behavioral responses [37,38]. Similarly to other components of fear, implicit avoidance can be acquired by associative learning and employed such that a threating forthcoming event is prevented (e.g. such as when someone with social phobia withdraws invitations to attend parties to avoid social embarrassment; [3,39]). Furthermore, when the fear of anxiety-inducing cues is generalized to stimuli that are perceptively similar to the original conditioned stimuli, individuals end up avoiding not only the conditioned stimuli but also a vast number of somehow related cues but otherwise harmless objects or situations [40]. Consequently, the presence of pervasive avoidance tendencies prevents individuals from experiencing the context as safe, hampering the success of fear extinction [41,42] by impairing new learnings and prompting to fear recovery.

**Fig 1:**
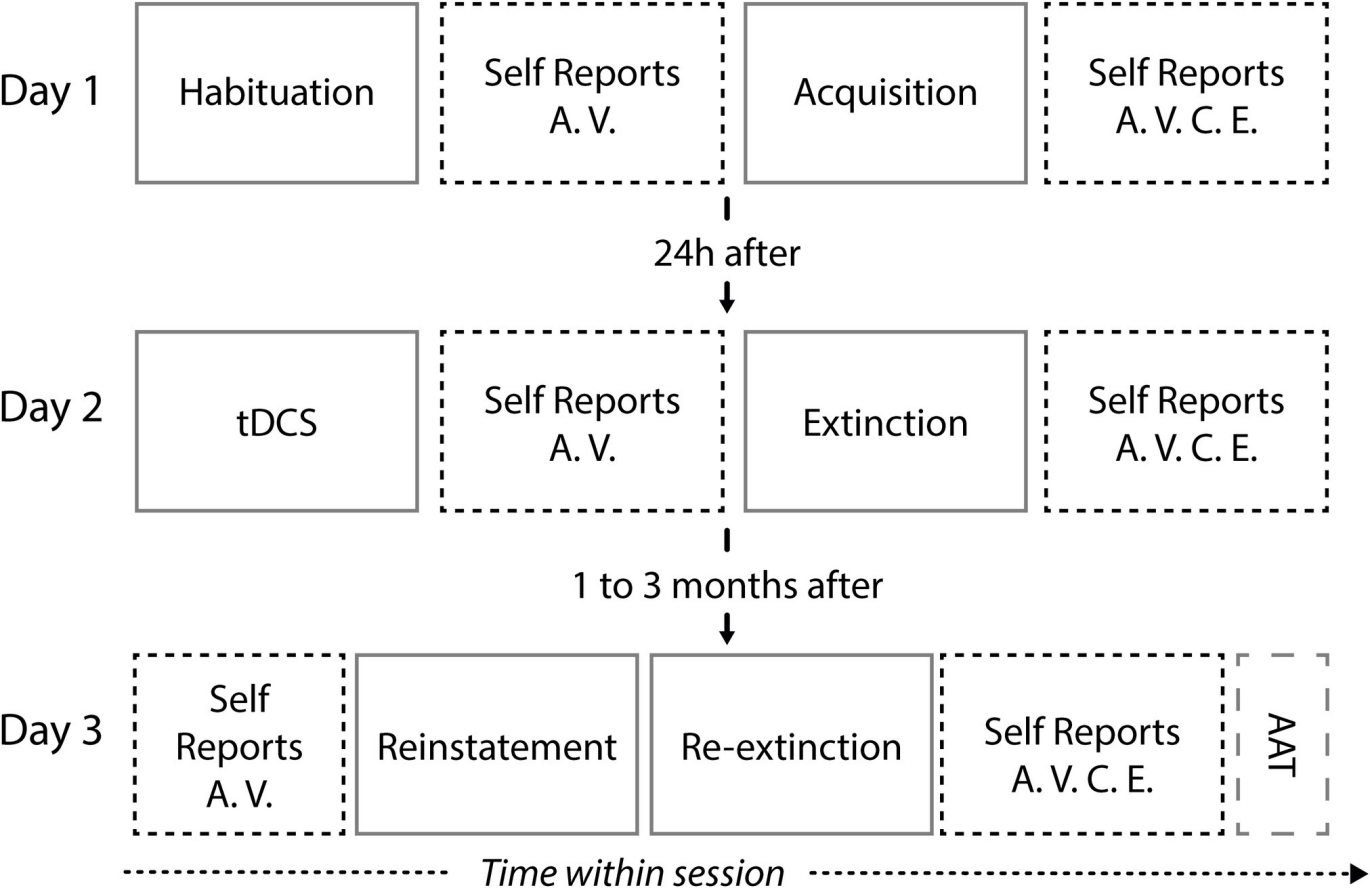
Experimental design. In day 1, participants went through habituation to stimuli (7 CS+; 7 CS-) and fear acquisition session (16 CS+, 16 CS-; 12 US). In day 2, participants were randomly assigned to the cathodal tDCS group or the sham tDCS group. The 20min tDCS session was followed by extinction training (16 CS+; 16CS-). In day 3, participants recovered the conditioned fear with a reinstatement procedure (4 US), followed by re-extinction (16 CS+; 16CS-) and the AAT (8 practice trials and 80 experimental trials). tDCS: Transcranial Direct Current Stimulation; AAT: Approach-avoidance Task; CS+: conditioned stimuli; CS-: unreinforced or control stimuli; US: Unconditioned stimuli. Self-reports: A–arousal; V–valence; C–contingency; E–expectancy.

In this study, we used a 3-day fear conditioning procedure to test whether cathodal tDCS over the rDLPFC enhances the efficacy of fear extinction procedures as assessed by three components of the fear response: the autonomic response (SCRs), the subjective experience (self-reports) and the implicit avoidance tendencies measured by reaction times (RTs; [40]; *cf*. Fig 1).

## Methods

After having been informed about the procedure and given written informed consent, a total of 48 women (mean age = 20.51 years, SD = 5.00) participated in the study. We tested only female participants due to known gender differences concerning electrodermal activity [32], and fear conditioning responses [43]. The study, including its experimental protocol, was conducted according to the Declaration of Helsinki and approved by the Ethical Committee of the Faculty of Psychology and Educational Sciences of the University of Coimbra (Ref. DIR352/2014). We defined the following exclusion criteria: < 18 years of age, history of psychiatric disorder and/or current psychoactive medication, screened by an experienced clinical psychologist using a short DSM-4-based clinical interview [44]; pregnancy; caffeine and/or alcohol intake 24h before sessions; having had any physical exercise or meal 2h before the start of each session [45]; auditory or visual (non-corrected) deficit; and contraindications to the use of tDCS [46]. Additionally, participants had to show contingency awareness, as measured by self-reported contingency ratings (CS+/US ≥ 50% and CS-/US < 50%; see “Self-report measures” section below).

### Stimuli

We used three stimuli–the unconditioned stimulus (US), the conditioned stimulus (CS+), and the neutral stimulus (CS-; [4, 16, 25]). The CS+ and CS- were 12x12cm squares, either blue or yellow presented against a white background for 4s (during habituation and acquisition sessions) or 16s (during extinction session). The presentation duration difference is due to this study being part of a broader project that includes fMRI data collection during extinction in a block design, which requires the stimuli to be presented longer to evoke the hemodynamic response and collect neural activity. The color assignment to the CS+ and the CS- was counterbalanced across participants. The US was a women’s scream (item 277 from the International Affective Digitized Sound System, [4,47]) delivered through noise-cancelling headphones, with intensity individually set between 90–96dB to correspond to the participants comfort level. US intensity was individually set in the first session, using a dummy aversive sound (item 276 from the International Affective Digitized Sound System; [44]) and a Visual Analog Scale for pain to measure discomfort [48].

We pseudo-randomized the CS+ and the CS- trials within each session of each day, such that no more than two consecutive presentations of the same category of stimuli were allowed. For stimuli presentation, we used E-Prime (2.0.10.353 Standard SP1, Psychology Software Tools, Pittsburgh, PA), connected to a DELLP2012H monitor.

### Fear conditioning procedure

The experiment consisted of a partial-reinforcement auditory fear conditioning procedure, conducted on two consecutive days (Day 1 –baseline psychological assessment questionnaires, fear habituation and fear acquisition; Day 2 –tDCS session and fear extinction) plus a follow-up session (Day3 –fear reinstatement, re-extinction and approach-avoidance task-AAT) one to three months later. For the cathodal group extinction was preceded by cathodal tDCS, whereas for the sham group, extinction was preceded by sham tDCS. During the 3 days, fear responses were measured by SCRs (except during tDCS stimulation and AAT), self-report ratings on valence, arousal, contingency and expectancy, and AAT (*cf*. Fig 1).

#### Day 1

Participants were randomly assigned to the cathodal or the sham group in a 2:1 ratio to compensate for cathodal stimulation variability (individual features that alter current flow and uptake and hinder tDCS polarity-specific results, such as hair thickness [49]). The habituation phase consisted of 8 non-reinforced presentations of the CSs. The acquisition phase consisted of a partial reinforcement procedure at 75%—i.e., 12 out of 16 presentations of the CS+ were paired with the auditory US. When the CS+ and US were paired, the presentation of the US overlapped with the last 2s of the CS+. The CS- was never paired with the US. In habituation and acquisition, stimuli were presented for 4s, followed by a jittered inter-stimulus interval (ISI; 10-12s), during the presentation of a black fixation cross over a white screen.

#### Day 2

Before the tDCS session, participants were asked to verbally recall the CS+. Between the tDCS session and extinction training the tDCS electrodes were removed from participants, SCRs electrodes were placed, participants verbally replied to an adverse effects questionnaire, and answered STAI-state scale and self-reports on the computer screen (taking 10 minutes maximum from the end of the stimulation session until the extinction training began).

The extinction training consisted of 16 CS+ and 16 CS- trials. Per trial, the fixation cross (ISI; 10-12s) was followed by the presentation of the CS+ or the CS- for 16s. The US was not presented during extinction.

#### Day 3

Participants were invited to a follow-up session one to three months later. Sessions started by asking participants to verbally recall the color of the CS+. The reinstatement phase consisted of four consecutive unsignaled USs for 2s each (jittered ISI = 1-20s). The re-extinction phase started immediately after reinstatement and was similar to the Day 2 extinction session.

### Transcranial Direct Current Stimulation (tDCS)

According to previous literature, at 1 mA, tDCS after-effects are expected to last between 40min [8] and 90min [50], as measured by motor evoked potentials or regional cerebral blood flow, respectively. The same literature suggests that after-effects are expected to out-last stimulation by means of long-term potentiation and long-term depression mechanisms. Accordingly, we set the tDCS session to be delivered offline for 20min long, with a constant current intensity of 1mA and .04mA/mm^2^ current density, delivered through a tDCS 1-channel stimulator (TCT Research Limited, Hong Kong). We placed the tDCS cathode electrode over the rDLPFC (F4), in accordance to the International 10–20 EEG System [51], and the anode electrode extra cephalically over the contralateral deltoid ([25]; *cf*. S1 and S2 Figs). We used two electrodes of 24.75cm^2^ wrapped in saline soaked sponges (0.9% sodium chloride). Current was ramped up and ramped down during the first and last 30s of stimulation [46]. At the end of the tDCS session, participants were instructed to report any adverse effects (*cf*. S1 Table).

### Self-report measures

Self-report measures were the main variables of interest (for a rationale see S1 File). We used Lang’s [52] Self-Assessment Manikin (SAM) scales to assess arousal and valence. Arousal ranged from one (“highly calm”) to nine (“highly excited”) and valence ranged from one (“highly unpleasant”) to nine (“highly pleasant”). We used a customized scale to assess contingency for the CSs/US association, from 0% (the CS was never paired with the US) to 100% (the CS was always paired with the US), in increments of 25%. To assess expectancy of the US presentation after each CS presentation, we used a customized scale ranging from zero (“I was sure the sound was not coming”), to nine (“I was sure the sound was coming”).

The rating scales were presented on the screen using E-Prime and answered using the keyboard.

### Psychological questionnaires

Day 1 baseline psychological assessment was answered via a computer screen, using a keyboard, and included the following instruments: Anxiety Sensitivity Scale –3-PT [53]; Behavioral Symptoms Inventory [54]; State Anxiety Inventory (STAI-1) and Trait Anxiety Inventory (STAI-2;[55]). On Day 2 and Day 3, participants gave their responses to STAI-1[55].

### Skin conductance responses

On Day 1, 2 and 3, Powerlab26T finger electrodes (MLT116F; ADInstruments, Ltd., Dunedin, New Zealand) were attached to the medial phalanges of the index and middle fingers of the left hand and connected to a galvanic skin response Amplifier (FE116; ADInstruments, Ltd., Dunedin, New Zealand). Data were collected at a rate of 5Hz, filtering out frequencies above 50Hz. The signal was pre-processed in MATLAB (2013, The MathWorks, Inc., Natick, Massachusetts, United States) using in-house scripts.

### Approach-avoidance task (AAT)

We used a symbolic task to assess implicit approach-avoidance components of fear [56]. We presented a manikin, either at the top or at the bottom of the screen, followed by the presentation of a white frame in the opposite position. The frame appeared for 2s, in a portrait or landscape position, and contained one of the CSs. Participants had to use the keyboard to move the manikin as fast as possible, towards or away from the frame, according to its orientation (explicit task-related response) and regardless the stimulus that was placed inside the frame (implicit fear-related response). Incorrect responses (when the participant response is not in agreement with the explicit task-related response) were followed by a red cross feedback presented for 0.5s; no responses would be followed by an attention-call after 13s. An ISI of 2s would follow participants’ response/feedback.

In this task, RTs are thought to be dependent on the relationship between the two independent types of responses: the explicit task-related response (i.e., to approach or avoid the frame according to its orientation), and the implicit fear-related response (*i*.*e*., to approach the neutral stimulus and to avoid the feared stimulus). If the action tendency component of the fear response is not fully eliminated (after extinction and re-extinction training procedures), there should be an effect of the implicit fear-related responses on the task-related approach/avoid responses. Specifically, RTs should be faster when the two independent types of responses are congruent (*i*.*e*., participants have to avoid the frame because of its orientation, and the frame contains the CS+), than when these are incongruent (*i*.*e*. participants have to approach the frame because of its orientation, and the frame contains the CS+; [38]). The procedure was adopted from the author’s previous works [56]. Each participant completed two blocks–one where they were instructed to move the manikin towards the frame when it was in landscape orientation, and another where participants were instructed to move the manikin away from the frame when it was in portrait orientation. The order of the blocks was counterbalanced across participants. The task included four practice trials per block using grey instead of colored squares. Each block had eight conditions, in a two (stimulus: CS+ vs. CS-) by two (frame orientation: portrait vs. landscape) by two (position of the frame: top vs. bottom) design and included a total of 40 experimental trials where we collected reaction times [39]. During the AAT, the headphones were not placed.

From the RTs we calculated the fear index, subtracting the RT for the fear-related response of avoidance from the fear-related response of approach for each CS [56]. A negative fear index mirrors the absence of avoidance tendencies, such that participants are faster to approach than to avoid, whereas a positive fear index shows the reverse tendency. We excluded incorrect trials and trials with response times bellow 200ms and above 3000ms and used median RTs to compute fear indexes per stimulus [56]. For an example of one AAT trial, see S3 Fig.

## Results

For the main analysis, we excluded 2 participants who did not successfully acquire a fear response according to contingency ratings. Three other participants were excluded due to missing data on Day 1 contingency (caused by a recording error). A total of 43 participants completed the experiment (mean age = 20.42 ± 4.99; mean education years = 13.00 ± 1.90), of which 27 were randomly assigned to the tDCS group and 16 to the sham group. Groups did not differ with respect to age, education and psychological questionnaires at baseline (*cf*. S2 Table). To perform the analysis, we applied Greenhouse-Geisser correction when the sphericity assumption was not fulfilled according to Mauchly’s Test.

**Fig 2:**
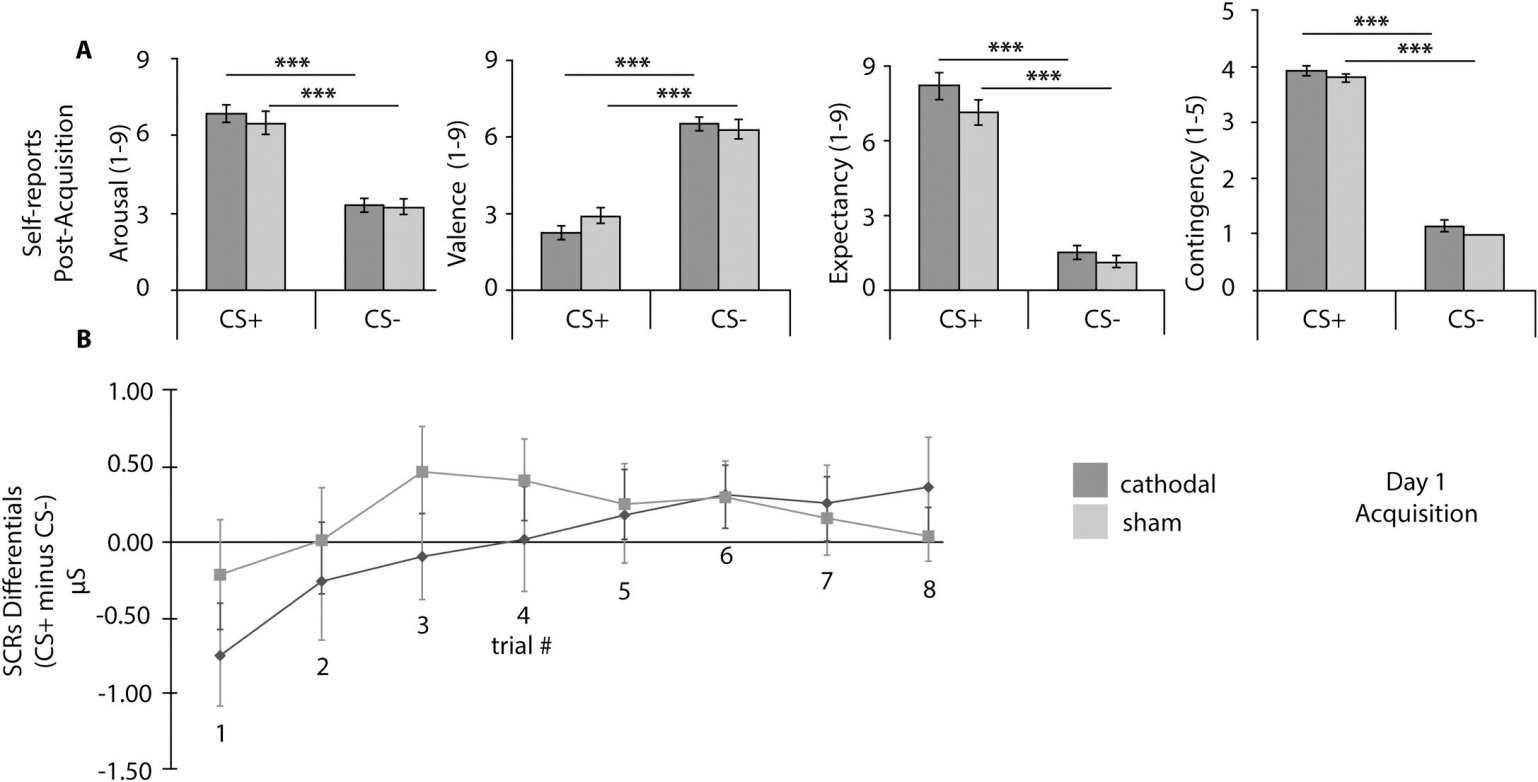
Day 1 Self-reports and SCRs differentials. A) Day 1 Post-Acquisition Self-reports of arousal, valence, expectancy and contingency. B) SCRs differentials (CS+ minus CS- per trial) for early phase (the first 8 trials). CS+: conditioned stimuli; CS-: non-reinforced or control stimuli; cathodal: tDCS cathodal stimulation group; sham: tDCS sham group. Error bars represent standard errors of the mean (SEM). * p < .05, ** p < .01, *** p < .001.

**Fig 3:**
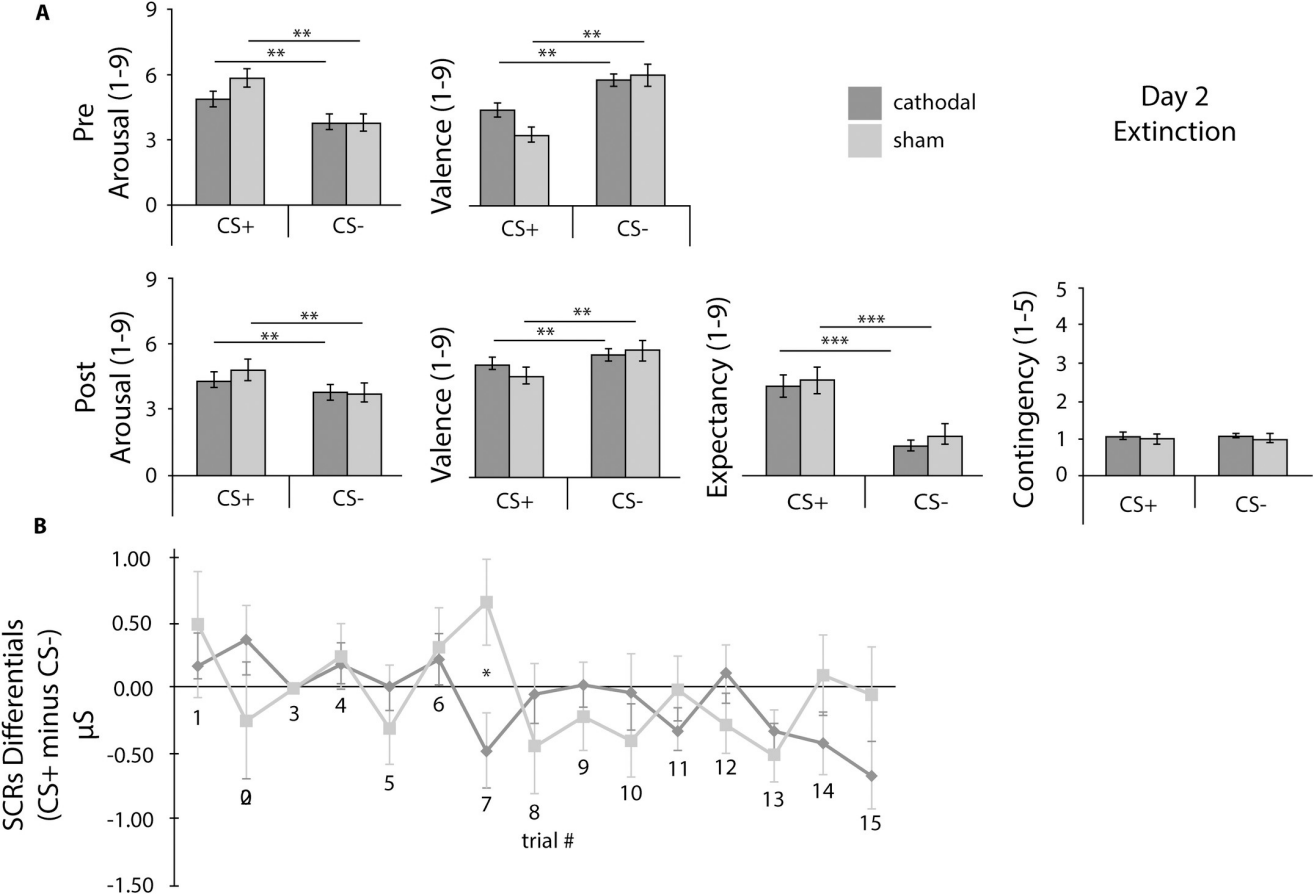
Day 2 Self-reports and SCRs differentials. A) Day 2 Pre- and Post-Extinction Self-reports of arousal, valence, expectancy and contingency. B) SCRs differentials (CS+ minus CS- per trial). CS+: conditioned stimuli; CS-: non-reinforced or control stimuli; cathodal: tDCS cathodal stimulation group; sham: tDCS sham group. Error bars represent standard errors of the mean (SEM). * p < .05, ** p < .01, *** p < .001.

**Fig 4:**
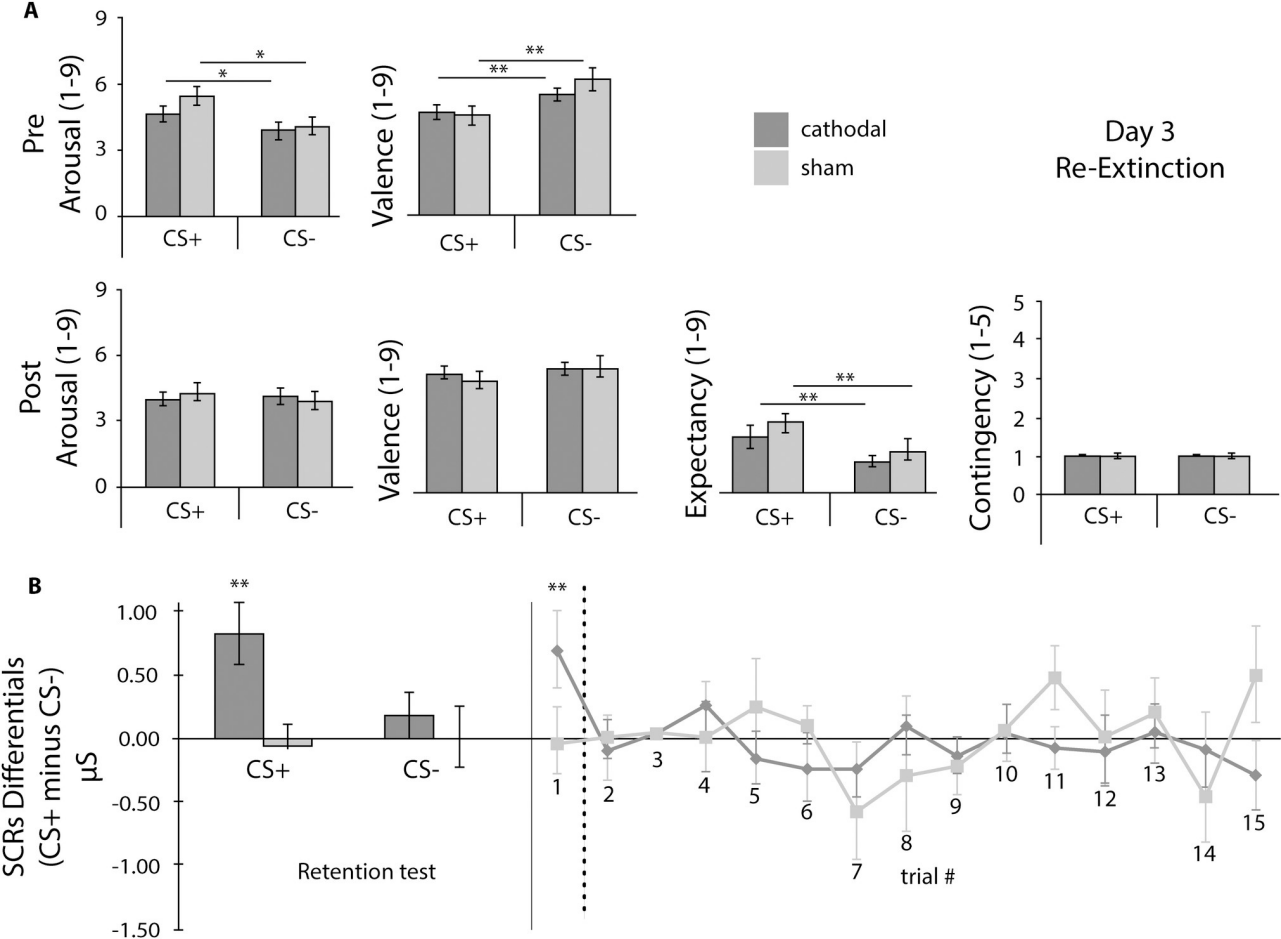
Day 3 Self-reports and SCRs differentials. A) Day 3 Pre- and Post-Re-Extinction Self-reports of arousal, valence, expectancy and contingency. B) Test phase for SCRs (for better visualization, the histogram depicts the test phase separately for the first CS+ and for the first CS-) and SCRs differentials (CS+ minus CS- per trial) for Re-Extinction. CS+: conditioned stimuli; CS-: non-reinforced or control stimuli; cathodal: tDCS cathodal stimulation group; sham: tDCS sham group. Error bars represent standard errors of the mean (SEM). * p < .05, ** p < .01, *** p < .001.

**Fig 5:**
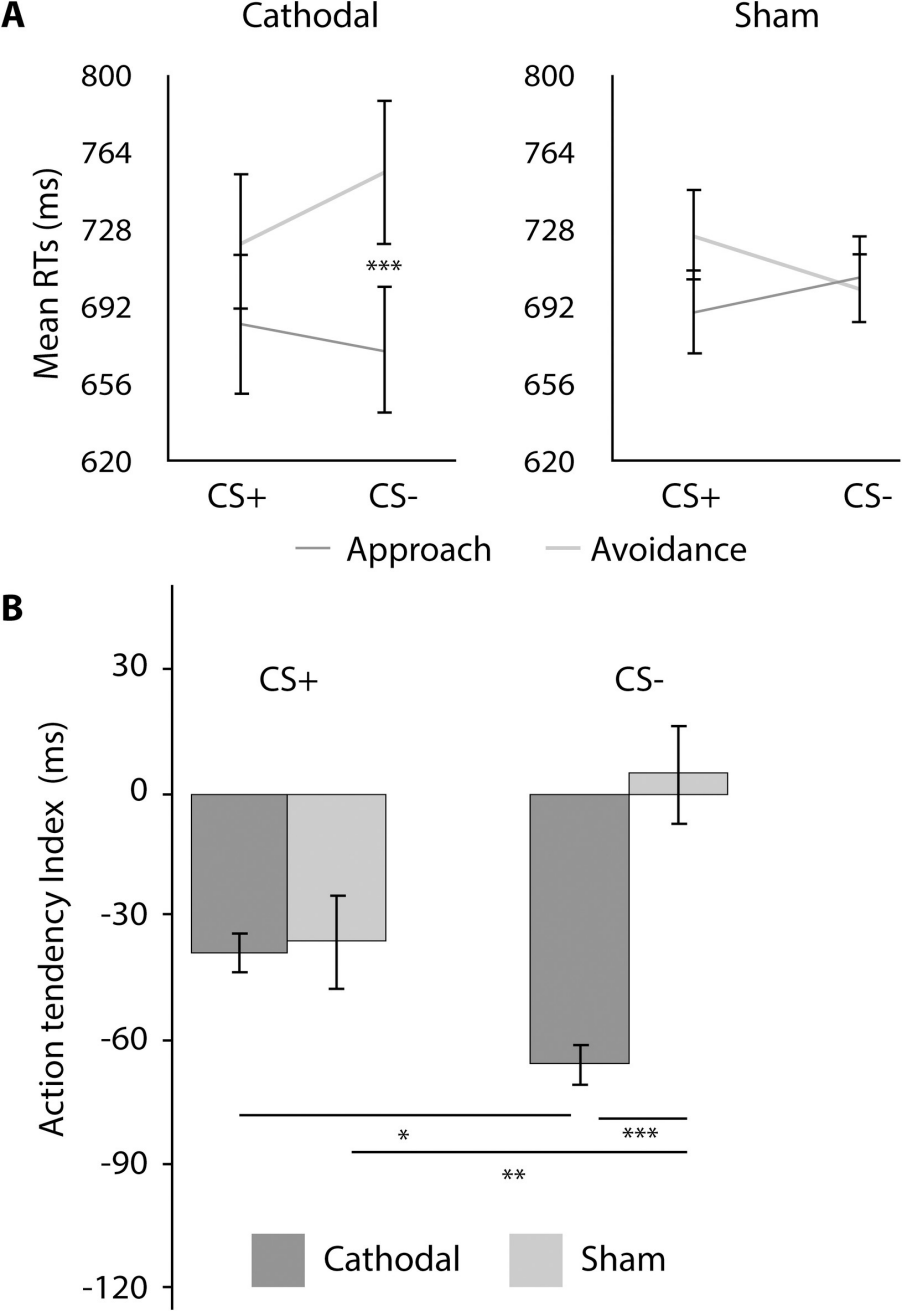
Approach-avoidance task (AAT) results. A) Approach and avoidance responses per experimental group. *** p < .001; CS+: conditioned stimuli; CS-: non-reinforced or control stimuli; cathodal: tDCS cathodal stimulation group; sham: tDCS sham group. Error bars represent standard errors of the mean (SEM).

Results will be described by testing day and supported by Table 1, and Figs 2–4 that summarize self-report ratings and SCRs differentials for Day 1–3. Additionally, Fig 5 depicts the results for the AAT for Day 3.

**Table 1:** Self-report ratings ANOVASs.

		Group x Stimuli				Group				Stimuli			
* *	Variable	df	F	η_p_^2^	p	df	F/U	η_p_^2^	p	df	F/Z	η_p_^2^	p
**Day1**	** **												
**habituation**	**Arousal**	1,41	.091	.002	.764	1, 41	.007	.000	.932	1, 41	2.822	.064	.101
	**Valence**	1,33	.005	.000	.942	1, 33	1.519	.044	.227	1, 33	1.162	.034	.289
**post**	**Arousal**	1,41	.279	.007	.600	1, 41	.257	.006	.615	1, 41	138.58	.772	.000***
**acquisition**	**Valence**	1,36	2.33	.061	.136	1, 36	.196	.005	.660	1, 36	120.77	.770	.000***
	**Expectancy**	1,20	.491	.024	.492	1, 20	3.26	.140	.086	1, 41	163.75	.891	.000***
** **	**Contingency**	1,41	.000	.000	.989	-	213.50	-	.936	1, 41	1240.89	.968	.000***
**Day 2**	** **												
**pre-extinction**	**Arousal**	1, 41	1.97	.046	.168	1, 41	1.43	.034	.238	1, 41	19.79	.330	.000***
**(post-tDCS)**	**Valence**	1, 41	3.13	.071	.084	1, 41	2.10	.049	.155	1, 41	29.97	.420	.000***
**post-extinction**	**Arousal**	1, 40	.64	.016	.427	1, 40	.104	.003	.749	1, 40	11.35	.220	.002**
	**Valence**	1, 41	2.01	.047	.164	1, 41	.296	.007	.589	1, 41	9.70	.190	.003**
	**Expectancy**	1, 40	.092	.002	.763	1, 40	.404	.010	.529	1, 40	52.12	.570	.000***
** **	**Contingency**	-	-	-	-	-	208.00	-	.441	-	.00	-	1
**Day 3**	** **												
**pre**	**Arousal**	1, 41	.645	.015	.427	1, 41	1.02	.024	.319	1, 41	7.18	.149	.011*
**re-extinction**	**Valence**	1, 41	1.046	.030	.313	1, 41	.673	.016	.417	1, 41	9.30	1.85	.004**
**post**	**Arousal**	1, 39	.39	.010	.536	1, 39	.019	.000	.891	1, 39	1.20	.030	.280
**re-extinction**	**Valence**	1, 41	.67	.016	.420	1, 41	.140	.003	.707	1, 41	2.26	.052	.141
	**Expectancy**	1, 41	1.71	.015	.440	1, 41	.563	.014	.458	1, 41	27.35	.400	.001**
** **	**Contingency**	-	-	-	-	-	216.00	-	1	-	.00	-	1

* < .05

** < .005

*** < .001

### Day 1- Habituation and fear acquisition

On day 1, *t*-tests on self-report measures suggested no evidence of between groups’ differences after habituation for the CS+ (arousal: t (41) = -.23, p = .818; valence: t (36) = 1.58, p = .123) and the CS- (arousal: t (41) = .07, p = .942; valence: t (38) = .48, p = .643). Similarly, there was no evidence of between groups’ differences after acquisition for the CS+ (arousal: t (41) = .72, p = .478; valence: t (36) = -1.50, p = .143; contingency: t (41) = .918, p = .364; expectancy: t (11.78) = 2.02, p = .084) and for the CS- (arousal: t (41) = .10 , p = .924; valence: t (36) = .34, p = .734; contingency: t (26) = 1.36, p = .185; expectancy: t(20) = .533, p = .600). The two-way repeated measures ANOVA with stimulus (CS+ or CS) as within-subjects factor, and group (cathodal or sham) as between-subject factor, also showed no interaction after habituation, and after fear acquisition. As expected, after fear acquisition there was a statistically significant main effect of stimuli for each of the four measures, confirming fear acquisition across groups and showing that the CS+ was rated as triggering increased arousal, evoking increased negative affect, which was perceived as more frequently paired with the US, and which led to increased expectancy of US presentations, when compared to the CS- (*cf*. Table 1).

Note that due to registration errors, we lost the post-acquisition expectancy ratings from fifteen participants of the cathodal group and six participants of the sham group. Notwithstanding the importance of expectancy ratings, this measure was not used to set fear-conditioning criteria, thus not critically influencing following procedures.

Independent samples *t*-tests for SCRs during the habituation and acquisition phases showed no differences between groups, per trial. Two-way repeated measures ANOVAs showed no interaction between groups and the trial order for SCRs differentials during habituation (F (6, 246) = .56, p = .762, η_p_^2^ = .013), and acquisition (F (8.993, 368,730) = .61, p = .787, η_p_^2^ = .015). No main effects were found during habituation for trial order (F (6, 246) = .80, p = .570, η_p_^2^ = .019) or for group (F (1, 41) = .28, p = .602, η_p_^2^ = .007); nor during acquisition for trial order (F (8.993, 368,730) = 1.29, p = .240, η_p_^2^ = .031) or group (F (1, 41) = .016, p = .901, η_p_^2^ = .000; *cf*. Fig 2).

### Day 2 –Fear memory extinction

ANOVA results on self-reports showed that after tDCS and before extinction, there was no interaction between stimuli and experimental group for arousal and valence. However, the main effect of stimuli due to fear acquisition was still present for arousal and valence, showing that despite the tDCS session, the CS+ was still evoking increased arousal and negative affect compared with the CS-. This result shows that there is no evidence for immediate cathodal tDCS effect as participants scored the stimuli in a similar way. After extinction, the interactions were still not statistically significant, the main effect of stimuli with increased fear-associated responses to the CS+ (of arousal, valence and expectancy) was still present, and no main effect of group was found. Regarding contingency ratings, the Wilcoxon Signed Rank Test, showed no main effect of stimuli, and the Mann Whitney test for group differences was not statistically significant. In sum, results show that, regardless of tDCS session, after extinction participants were able to correctly identify the CS+ and report the absence of the aversive stimulus during extinction, both showing intact declarative associative memory CS+/US (expectancy ratings), the estimation of risk for the CS+ and for the CS- was equivalent (contingency ratings), showing fear extinction. However, as there were no between group differences, there is no evidence for short-term cathodal tDCS effect over extinction (*cf*. Table 1).

As for SCRs, no interaction was found between group and trial order (F (14, 574) = 1.36, p = .166, η_p_^2^ = .032) nor were there any main effects of group (F (1, 41) = .87, p = .355, η_p_^2^ = .021). Nevertheless, as expected, there was a main effect of trial order due to the extinction procedure (F (14, 574) = 1.76, p = .041, η_p_^2^ = .041). Results suggest that there were no short-term differences between groups along extinction, in what concerns the autonomic fear response (*cf*. Fig 3).

### Day 3 –Reinstatement and re-extinction

Participants were tested 1 to 3 months after extinction. After recall but before re-extinction, the ANOVA results were similar to Day 2 post extinction, with no interaction between stimuli and experimental group for arousal or valence. Also, the main effect of stimuli was still present for arousal and valence, and no main effect of group was found. The same happened after extinction recall and re-extinction with no statistically significant interaction between group and stimuli for arousal, valence, and expectancy. The main effect of stimuli present on Day 2, disappeared after re-extinction for arousal, valence and contingency but not for expectancy, indicating equivalent probability estimates of risk for the CSs despite the ability to explicitly discriminate the CSs. Again, no main effect of group was present for arousal, valence, expectancy or contingency (*cf*. Table 1).

Using SCRs, we tested *acquisition/extinction retention* with two-way repeated measures ANOVAs having stimuli (the first CS+ and the first CS- presentation) as within subject factor, and group (cathodal or sham) as between subjects factor, and we found no statistically significant interaction (F (1, 41) = 2.57, p = .117, η_p_^2^ = .059) and no main effect of stimuli (F (1, 41) = 1.67, p = .203, η_p_^2^ = .039). However, we found a significant main effect of group (F (1, 41) = 5.24, p = .027, η_p_^2^ = .113), with the cathodal group showing increased SCRs to both the CSs, but particularly to the CS+. This result showed that tDCS may have a detrimental effect in augmenting long-term fear savings. However, when running a group by trial (2x15) repeated measures ANOVA across the re-extinction session, no interaction was found (F (9.586, 393.025) = 1.0, p = .445, η_p_^2^ = .024), and no main effects were present for the trial order (F (9.586, 393.025) = .89, p = .537, η_p_^2^ = .021) or for group (F (1, 41) = .388, p = .537, η_p_^2^ = .009), suggesting no evidence of cathodal tDCS impact on SCRs across the re-extinction process (*cf*. Fig 4). We further calculated a simple linear regression to predict SCRs in extinction (late phase [last 5 trials]) controlling for the number of weeks between session 2 and session 3 per group. We found that the time window between session 2 and session 3 did not significantly impact SCRs either for the tDCS group (F (1, 22) = 1.00, p = .328; R^2^ = .04) or for the sham group (F (1, 14) = .599, p = .452, R^2^ = .04).

On Day 3, participants also completed the AAT, from which we excluded the incorrect trials (6.83%) and trials with response times below 200ms and above 3000ms (4.30%). To explore differences in AAT performance across groups we ran a three-way repeated measures ANOVA with stimuli (CS+ and CS-) and response (approach and avoidance) as within-subjects factor and group (cathodal and sham) as between-subjects factor.

As expected, the three-way interaction was statistically significant (F (1, 41) = 5.97, p = .019, ηp^2^ = .127), meaning that the three factors contribute to the RTs. However, the between subjects’ tests show no differences between groups (F (1, 41) = .002, p = .962, ηp^2^ = .000). As such, to further explore the data, we followed up with two-way repeated measures ANOVAs using stimuli and response as within-subjects’ factors, for each group separately. We found a significant cross-over interaction for the sham group (F (1, 15) = 4.72, p = .046, ηp^2^ = .239) but not for the cathodal group (F (1, 26) = 3.34, p = .079, ηp^2^ = .114). That is, for the sham group, the effect of the stimuli differs with the type of response, although no main effects were found (differences between stimuli in avoidance RTs: t (15) = 1.39, p = .185; 95% CIs [-13.55, 64.43]; differences between stimuli in approach RTs: t (16) = -.76, p = .460; 95% CIs [-59.58, 28.33]). For the cathodal group there were no significant results for the two-way ANOVA. However, we further explored the responses in a similar manner to the sham group. Follow-up planned contrasts for the cathodal group revealed a marginal difference between the avoidance RTs to the CS+ and the avoidance RTs to the CS- (t (26) = -2.00, p = .056; 95% CIs [-66.86, .97]), which did not survive corrections for multiple comparisons (t (26) = -2.00, p = .28; 95% CIs [-66.86, .97]). Furthermore, for the cathodal group we found a difference between the approach and the avoidance responses to the CS- (t (26) = -4.86, p < .0001, 95% CIs [-118.96, -47.89], Bonferroni corrected) with overall faster approach RTs, suggesting a positive valence attributed to the CS- (two-tailed; Fig 5A).

Following previous literature [40], we computed the avoidance tendency index (the time to approach subtracted from the time to avoid) for each stimulus, in each group. Two-way repeated measures ANOVA, with the stimuli (CS+, CS-) as within-subjects factor, and the group (cathodal, sham) as a between-subjects factor showed an interaction between stimuli and experimental group with a moderate effect size (F (1, 41) = 5.97, p = .019, ηp^2^ = .127). Univariate analyses suggest that whereas there was no difference between groups in terms of the avoidance tendency towards the CS+ (F (1, 41) = .006, p = .937, ηp^2^ = .000), there was indeed a difference between groups in the avoidance tendency towards the CS- (F (1, 41) = 12.04, p = .001, ηp^2^ = .227). In fact, a moderate effect size is present for the estimated difference between groups, with the avoidance tendency presented by participants of the cathodal group towards the CS- following the trend of positive stimuli (positive bias), whereas for the sham group it remains equal to zero (*cf*. Fig 5B). Although caution is required for the interpretation of the results in the absence of an interaction between stimuli and response, this result suggests that whereas tDCS may have no long-term effect over the avoidance tendency towards the conditioned stimuli, it may have an effect over the avoidance tendency towards the neutral stimuli, leading to a disambiguation of its value.

## Discussion

In this study, we assessed whether cathodal tDCS over the rDLPFC enhances the efficacy of fear extinction procedures across three distinctive components of the fear response–the autonomic response (SCRs), the subjective experience (self-reports) and the implicit avoidance tendencies (AAT index).

In a sample of 34 women, we found no evidence of cathodal tDCS short-term impact in the way participants perceive threatening cues, according to its autonomic component. Surprisingly, offline cathodal tDCS shows a long-term negative effect on SCRs, as illustrated by our acquisition/extinction retention test, wherein participants in the cathodal group showed increased fear memory savings to the conditioned stimulus (for a discussion concerning the definition of retention test, see Lonsdorf et al., 2019; [57]). Abend et al. [23], within a similar procedure aimed at disrupting reconsolidation and found a comparable result using low-frequency AC to produce long-term depression effect (LTD). Like AC, cathodal tDCS is expected to decrease neural activity by depolarizing apical dendrites and hyperpolarizing the somatic regions of the pyramidal cells under the cathode electrode [58]. Together, these results show no evidence of effect for cathodal tDCS over short-term fear conditioned SCRs, even suggesting a detrimental effect of cathodal tDCS over long-term fear and SCRs.

However, both cathodal tDCS and AC, result in the preservation of adequate stimuli discrimination, whereas anodal stimulation leads to a fear generalization effect to the CS- [19,23]. In generalization, the conditioned fear response spreads to similar perceptual stimuli. Subsequently, same class stimuli become aversive and individuals learn that they need to be avoided. In our study, we confirm previous literature findings concerning the use of AC [23] and show that across measures, fear generalization does not occur after cathodal stimulation, thus strengthening the hypothesis that the generalization effect is a polarity adverse outcome exclusive of anodal stimulation.

Furthermore, our study shows how different fear measures may suggest distinctive conclusions. Whereas affective, contingency, expectancy and self-reported state anxiety show no effect, SCRs suggest increased long-term fear memory savings for the CS+, after cathodal stimulation. This asynchrony was previously linked to the lack of overlap between the mechanisms behind each measure. In fact, the expectancy of harm or the intolerance to uncertainty that emerges during the extinction procedure by the absence of the US are differently translated by explicit ratings and SCRs [33,59–61]. In our study, the self-reported contingency (contingency learning) is congruent with successful extinction. However, the self-reported expectancy of the US (risk estimation) is still present after extinction, and the SCRs retention test shows a perseverative fear conditioned memory.

Finally, our results on avoidance tendencies partially support SCRs data in that the tendency to avoid the CS+ has decreased after classical extinction with or without cathodal tDCS. However, the way cathodal tDCS benefits extinction, do not occur by decreasing the fear response towards the threatening cue, but through the mitigation of the generalization effect to similar stimuli. This means that although cathodal stimulation has no direct effect on the new learning about the CS+, it may have an effect on the CS-. Indeed, we see that for the cathodal group there seems to be a positive bias towards the CS-, which is not present in the sham group. On the contrary, for the sham group, the avoidance tendency to the neutral cue increased beyond what would be expected. According to the AAT index, it seems then that tDCS may indeed have an effect on safety learning and on neutralizing the fearful response pattern to both threat cues and neutral cues, limiting generalization.

A generalization effect of the fear response to the CS- was previously found as a result of anodal tDCS [19,23], and our study suggests that this effect may be polarity-dependent. If cathodal stimulation is effective in reducing anxiety symptoms in case reports [25,26], the same effective response would be expected with respect to boosting its analogous experimental extinction procedures. Indeed, although there is a detrimental effect of cathodal stimulation on extinction according to SCRs, there seems to be a beneficial effect of cathodal stimulation in relation to the stimuli discrimination process associated with implicit action tendencies. Accordingly, limiting generalization to perceptively similar stimuli can be the mechanism by which cathodal tDCS succeeds in mitigating anxiety-related symptoms.

Here we show that cathodal stimulation of the rDLPFC enhanced the safety learning about similar neutral cues (the CS-). As the previous literature states, this may be so by directly decreasing the DLPFC activity, which is known to respond to stimuli similar to the CS+ [60], or by indirectly enhancing the vmPFC response to the CS- during extinction [62]. Regardless of the actual mechanism of action, our study suggests that cathodal tDCS may be altering simultaneous activity of a whole circuit, interfering with the balance between excitation and inhibition of different regions within the fear network, thus boosting discriminative processes [40].

A single cathodal tDCS session may indeed impact both short- and long-term extinction (1 to 3 months) and the fear system components. According to the literature, cathodal tDCS of 1mA can lead to both immediate and prolonged polarity-specific effects [8]. Whereas the immediate effects of tDCS are due to the modulation of the membrane resting potential (by affecting ion channels and altering its homeostasis), the after-effects rely on the modulation of enduring synaptic plasticity, interfering with long-term potentiation (LTP) and long-term depression (LTD). These are activity-dependent plasticity mechanisms that result in persistently enhanced or reduced synaptic transmission. Furthermore, the effect of tDCS on LTP and LTD is triggered by prolonged stimulation durations (such as a single 20min session) and is also moderated by concurrent task-specific synaptic plasticity [14]. That is to say, the tDCS effect is task-dependent in that its effect is moderated by concurrent behavioral interventions such as associative learning (of which fear conditioning is one example). Particularly, and as already shown in animal models, the combination of cathodal tDCS with behavioral fear conditioning modifies the strength of the associative learning by changing the functional properties of the network [63].

Furthermore, we know that tDCS modulates long-distance connectivity to subcortical structures [11]. Hence, previous fMRI studies show that the DLPFC downregulates fear responses through projections to the vmPFC, which in turn inhibit the amygdala during extinction learning. However, how the tDCS affects the amygdala is still not clear. Previous literature has shown increased vmPFC activity during classical extinction [17] but decreased activity when extinction occurs within the reconsolidation window [18]. Whereas in our study we found that cathodal tDCS, putatively occurring during the reconsolidation window may reduce the fear index/avoidance tendency in the long-term, Mungee et al. [24] detected no effect for cathodal tDCS in SCRs. Whilst in our study we asked participants to verbally recall the CS+ followed by classical extinction (Day 2 and 3), in Mungee et al. [24] there was no information update because participants did not go through a complete extinction procedure following tDCS. However, according to seminal studies, this new information is a necessary condition for the update of the CS+/US association [27].

On the other hand, the atypical reminder we used seemed to be less effective in triggering reconsolidation, and the expected update of the associative learning concerning the CS+ was not achieved (see the increased fear recovery at the beginning of Day 3, indexed by SCRs). Also, whether cathodal tDCS has successfully modulated reconsolidation boundaries concerning the conditioned fear response towards the CS- remains an open question in the absence of a factorial design. Moreover, the claim of decreasing the vmPFC participation during extinction using cathodal tDCS is not straightforward in the absence of neuroimaging data. In this sense, although our results may further contribute to the field about the usefulness of tDCS in boosting extinction procedures, it does not inform the recent debate surrounding reconsolidation theory robustness and consistency when applied to fear conditioning procedures in humans [64,65].

It is worth to mentioning that the short time window of interest that we defined for SCR data processing (i.e., 3s), may be the reason why we were not able to find an effect of trial order during the acquisition phase. Importantly, we were able to find a trial order effect during extinction phase where the duration of presentations were three times longer. To set a 3s window we relied on prime literature about SCRs processing [32], according to which a window of 1-3s after stimuli onset seems to be adequate across most studies and paradigms. However, because SCRs are slow autonomic responses, this short window may also be conservative failing to fully depict the values found in fear conditioning paradigms, particularly during acquisition phase. Indeed, a recent review by Londsdorf et al [33] recommend an extended window between 1s-4s.

Additionally, our experimental groups had unequal sample sizes to compensate for the variability driven by the individual differences. That is, features such as cortical and hair thickness are known to interfere with the tDCS current flow and considered to hinder tDCS polarity-specific results [66]. The downside of this option comes with reducing statistical power driven by the smaller group (n = 16); [67].

In sum, results seem to suggest that after cathodal tDCS, safety learning is enhanced, according to action tendencies, with boosted discrimination between threatening and neutral cues and a positive bias towards potentially ambivalent stimuli is present in the implicit behavioral fear system. This transference of positive affect is illustrated not only by the absence of avoidance tendencies towards the fear-conditioned stimulus (CS+), but also by a positive bias towards the neutral stimulus (CS-) on Day 3, only present for the cathodal group.

We used tDCS to boost extinction and to persistently eliminate conditioned fear responses. Although cathodal tDCS does not bring any particular improvement to declarative memory associated measures (self-reports and SCRs), the generalization of the implicit behavioral fear response to perceptively similar stimuli seems to be achieved. As generalization is a known transdiagnostic factor across anxiety disorders, and one of the hardest features to overcome in therapy, the beneficial potential of cathodal tDCS in increasing safety learning must be further explored along with anodal tDCS as an add-on enhancer of fear extinction memory.

## Supporting information

S1 Fig tDCS montage. The cathode electrode (represented by a minus sign) was positioned over F4 (i.e., right dorsolateral prefrontal cortex). The anode electrode (represented by a plus sign) was positioned over the left deltoid muscle; cz = vertex.
(TIF)

S2 Fig COMETS2 computational head model of electric field due to 1 mA cathodal tDCS. Head model of computational current flow using 3D finite element method to electric current conduction analysis in COMET2, for a tDCS montage at 1 mA, where the cathode is positioned over the F4 and the anode positioned right bellow the T5. The highest current density is found at the borders of the electrode pads.
(TIF)

S3 Fig AAT trial example. Time-line for a trial example of the AAT.CS+: conditioned stimuli; CS-: non-reinforced or control stimuli.
(TIF)

S1 Table Reported tDCS stimulation adverse effects. Self-reported adverse effects after 20-min tDCS session per experimental group. tDCS cathodal stimulation group; sham: tDCS sham group.
(DOCX)

S2 Table Statistics at baseline for socio-demographic and psychological assessment variables. Mean values and standard errors of the mean (SEM) for self-reported socio-demographic data and psychological assessment scores per experimental group. US: Unconditioned stimuli; ASI-3-PT: Anxiety Sensitivity Scale Portuguese version; BSI (GSI): Global index for symptoms intensity of the Behavioral Symptoms Inventory; STAI 1: State Anxiety Inventory; STAI 2: Trait Anxiety Inventory.
(DOCX)

S1 File Supplementary information. This document includes supplementary text about methods (participants, measures, data collection and pre-processing data, and analytic strategy, results about adverse effects, and psychological questionnaires) and references.
(DOC)
