# Supplementary figures and images for: The effect of cathodal tDCS on fear extinction: A cross-measures study
Source: PLoS One. 2019 Sep 18;14(9):e0221282. doi: 10.1371/journal.pone.0221282 (PMC6750569; doi:10.1371/journal.pone.0221282)

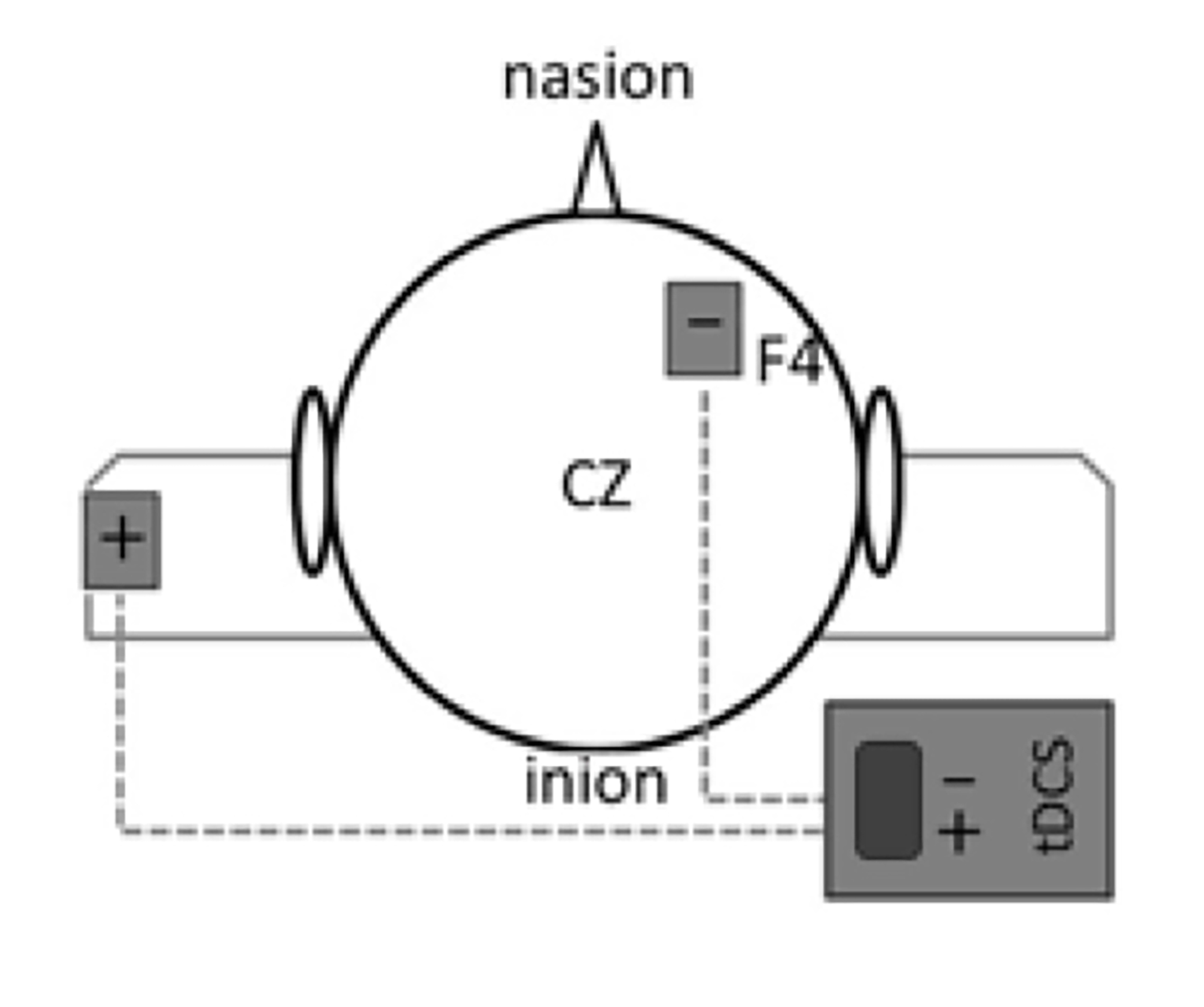

Supplement: S1 Fig — The cathode electrode (represented by a minus sign) was positioned over F4 (i.e., right dorsolateral prefrontal cortex). The anode electrode (represented by a plus sign) was positioned over the left deltoid muscle; cz = vertex. (TIF) [file pone.0221282.s001.tif]

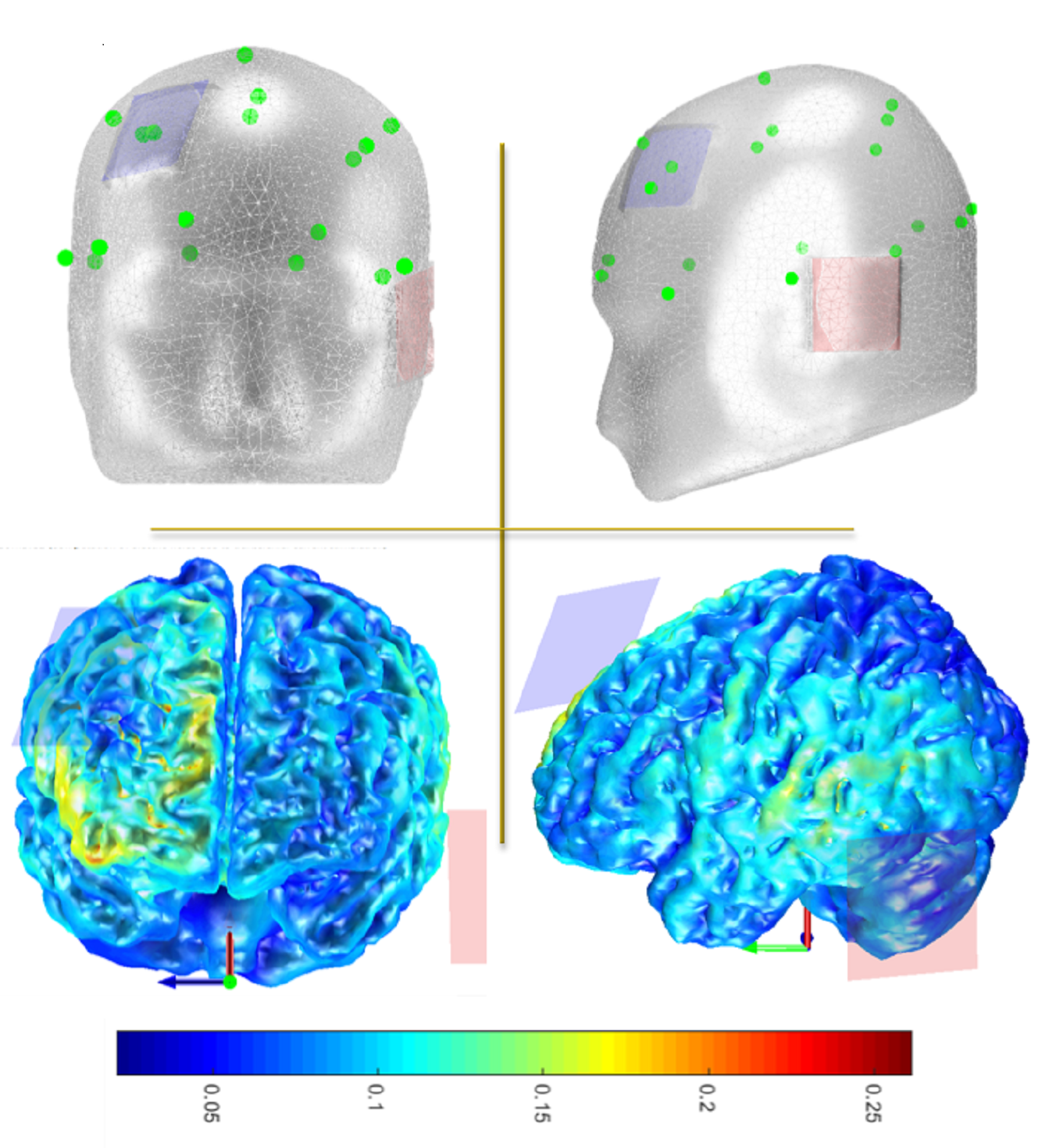

Supplement: S2 Fig — Head model of computational current flow using 3D finite element method to electric current conduction analysis in COMET2, for a tDCS montage at 1 mA, where the cathode is positioned over the F4 and the anode positioned right bellow the T5. The highest current density is found at the borders of the electrode pads. (TIF) [file pone.0221282.s002.tif]

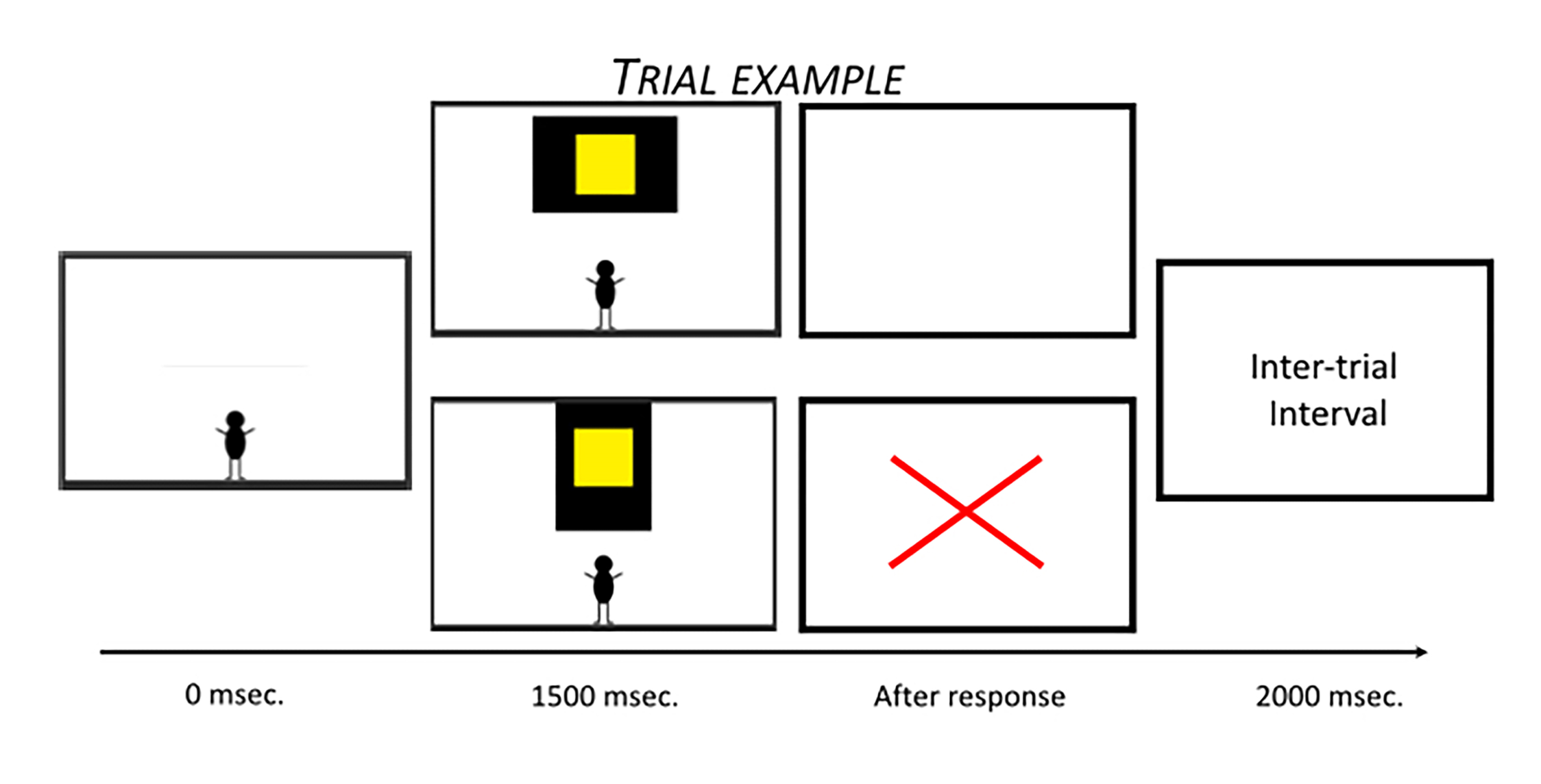

Supplement: S3 Fig — Time-line for a trial example of the AAT.CS+: conditioned stimuli; CS-: non-reinforced or control stimuli. (TIF) [file pone.0221282.s003.tif]
