# Supplementary material for: The effect of cathodal tDCS on fear extinction: A cross-measures study
Source: PLoS One. 2019 Sep 18;14(9):e0221282. doi: 10.1371/journal.pone.0221282 (PMC6750569; doi:10.1371/journal.pone.0221282)
Supplement: S1 Table — Self-reported adverse effects after 20-min tDCS session per experimental group. tDCS cathodal stimulation group; sham: tDCS sham group. (DOCX) [file pone.0221282.s004.docx]

**S1 Table**. *Reported tDCS stimulation adverse effects.*

|  | **Cathodal** | **Sham** | **p** |
| --- | --- | --- | --- |
|  | n = 27 | n = 16 |  |
| **Headache** | 3 | 0 | .172 |
| **Neck pain** | 0 | 0 | 1 |
| **Scalp discomfort** | 0 | 0 | 1 |
| **Tingling** | 8 | 2 | .204 |
| **Itching** | 5 | 0 | .070 |
| **Bruning sensation** | 1 | 1 | .705 |
| **Local skin redness** | 5 | 0 | .070 |
| **Somnolence** | 7 | 2 | .301 |
| **Attention deficit** | 1 | 0 | .441 |
| **Mood changes** | 0 | 1 | .194 |
| **Others** | 1 | 0 | .441 |

Note. Frequencies and significance *p* value from Mann-Whitney U for independent samples are depicted. Cathodal: tDCS cathodal stimulation group; Sham: tDCS sham group.
