# Supplementary material for: The effect of cathodal tDCS on fear extinction: A cross-measures study
Source: PLoS One. 2019 Sep 18;14(9):e0221282. doi: 10.1371/journal.pone.0221282 (PMC6750569; doi:10.1371/journal.pone.0221282)
Supplement: S2 Table — Mean values and standard errors of the mean (SEM) for self-reported socio-demographic data and psychological assessment scores per experimental group. US: Unconditioned stimuli; ASI-3-PT: Anxiety Sensitivity Scale Portuguese version; BSI (GSI): Global index for symptoms intensity of the Behavioral Symptoms Inventory; STAI 1: State Anxiety Inventory; STAI 2: Trait Anxiety Inventory. (DOCX) [file pone.0221282.s005.docx]

**S2 Table**. *Statistics at baseline for socio-demographic and psychological assessment variables.*

|  | **Cathodal** | **Sham** | **p** | **df** |
| --- | --- | --- | --- | --- |
|  | n = 27 | n = 16 |  | |
| **Age, Years** | 19.81 (2.56) | 21.44 (7.53) | .416 | 17.08 |
| **Education, Years** | 13.00 (1.78) | 13.00 (2.13) | 1.00 | 41 |
| **US Intensity, %** | 93.34 (3.50) | 92.07 (4.20) | .290 | 41 |
| **ASI-3-PT** | 26.93 (13.09) | 21.25 (10.97) | .153 | 41 |
| **BSI (GSI)** | .03 (.01) | .03 (.01) | .492 | 40 |
| **STAI 1** | 2.34 (.32) | 2.32 (.19) | .771 | 41 |
| **STAI 2** | 2.42 (.20) | 2.44 (.28) | .815 | 40 |

Note. Mean values are depicted. The standard errors of the mean (SEM) are within parenthesis. US: Unconditioned stimuli; ASI-3-PT: Anxiety Sensitivity Scale Portuguese version; BSI (GSI): Global index for symptoms intensity of the Behavioral Symptoms Inventory; STAI 1: State Anxiety Inventory; STAI 2: Trait Anxiety Inventory. Cathodal: cathodal stimulation group; Sham: tDCS sham group.
