# Supplementary material for: The effect of cathodal tDCS on fear extinction: A cross-measures study
Source: PLoS One. 2019 Sep 18;14(9):e0221282. doi: 10.1371/journal.pone.0221282 (PMC6750569; doi:10.1371/journal.pone.0221282)
Supplement: S1 File — This document includes supplementary text about methods (participants, measures, data collection and pre-processing data, and analytic strategy, results about adverse effects, and psychological questionnaires) and references. (DOC) [file pone.0221282.s006.doc]

Supplementary Information for:

The effect of cathodal tDCS on fear extinction: a cross-measures study

Ana Ganho-Ávila^1,2*^, Óscar F. Gonçalves^2,3,5^, Raquel Guiomar^1^, Paulo Sérgio Boggio^6^, Manish Kumar Asthana^6^,^7^, Angelos-Miltiadis Krypotos^8^, Jorge Almeida^1,4^

* Correspondence concerning this article should be addressed to:

Ana Ganho-Ávila,

Proaction Laboratory,

Faculty of Psychology and Educational Sciences,

University of Coimbra,

3001-802 Coimbra, Portugal.

Email: ganhoavila@fpce.uc.pt

Data collection protocol is available at: <https://osf.io/rmnq3/?view_only=1a9110a2a178453789df74c885a995b6>

This document includes:

- Supplementary Text

Methods

Participants

Measures, data collection and pre-processing data

Analytic strategy

Results

Adverse effects

Psychological questionnaires

- References

## Methods

**Participants**

In this study, we tested only female participants due to known gender differences concerning electrodermal activity [1], and fear conditioning responses [2].

## Measures, data collection and pre-processing data

Previous literature shows that SCRs may not reliably offer a measure of fear extinction [3]. In fact, according to our own experience as well as the experience from other colleagues, the amplitude of SCRs decreases with stimuli repetition which may lead to apparent extinction learning when in fact there is only habituation to stimuli.

Accordingly, self-report measures of fear learning (valence, arousal, expectancy and contingency) were used as the main variables of interest for this study, offering both the affective and the cognitive components of the fear response. In our study, we were particularly interested in contingency ratings, defined a subjective measure of CSs discrimination. Indeed, we used contingency ratings as an adequate performance-based criterion, to establish successful acquisition and extinction of fear [3]. Expectancy ratings on the other hand, are defined as the individual’s estimation of risk – the probability estimates of being presented with the aversive unconditioned stimulus (US) in future trials [4]. Because overestimations of risk is associated with the etiology of anxiety disorders, the use of contingency ratings can be considered to have high predictive value of the conditioned fear response.

**Skin conductance responses**

To protect for device drifts, we performed pre- baseline correction using the mean value of the 0.5s immediately before stimuli onset [5]. Because data was not normally distributed, we z-transformed SCR values [6]. We adopted the last observation carried forward imputation method for missing values due to artifacts, as this is the most conservative option. We kept only participants for which the total number of artifacts were less than half of the total trials per stimulus. SCRs analyses were conducted over computed CSs differentials between CS+ and CS- per trial. The CS- was always set to be the first stimulus to be present in each session and it was disregarded, as it was assumed to be an orientation response [7]. To even the number of trials for each CSs we disregarded the last CS+ within each session. We calculated SCRs on a trial-by-trial basis using data from the first 3s after the onset of each stimulus.

## Analytic strategy

## Psychological questionnaires

## To discard baseline differences that could otherwise influence results, we used independent *t*-tests to compare groups concerning each psychological measure. Further, independent samples *t*-test were used to observe tDCS impact over state anxiety, comparing group scores before extinction (day 2) and reinstatement (day 3).

## Self-reports

To make sure of the equivalency of groups at baseline we ran independent sample *t*-tests for the affective ratings of arousal and valence after habituation, and for arousal, valence, contingency and expectancy after acquisition. To understand the effects of stimulus and experimental group over self-report ratings, we performed a two-way repeated measures ANOVA with stimulus (CS+, CS-) as within-subjects factor, and group (cathodal, sham) as between-subject factor for day 1 (post-acquisition), day 2 (pre- and post-extinction) and day 3 (pre- and post-re-extinction).

**Skin conductance responses.**

In order to confirm similarity between groups during habituation and acquisition phases, we ran independent samples *t*-tests per trial. To better understand fear, as indexed by the SCRs during acquisition (day 1), extinction (day 2), and re-extinction (day 3), we performed two-way repeated measures ANOVAs for the CSs differentials, with trial (1 to 15) as within-subjects factor, and group (cathodal, sham) as between-subject factor. To observe fear recovery after reinstatement in day 3, we computed an independent samples *t*-test for the first responses to the CS+.

Approach-avoidance task (AAT)

Three-way repeated measures ANOVA followed by simple contrasts allowed us to understand the interaction between stimulus (CS+ or CS-) and fear related response (approach or avoidance) as within subject factors, and tDCS group (cathodal or sham) as the between subject factor. In case of no interactions we further explore the data using simple contrasts to better understand within and between group patterns. Following previous literature [8], we further ran a two-way mixed ANOVA for the fear index followed by univariate analysis.

**Results**

## Adverse effects

As expected from previous literature, participants reported few mild adverse effects to tDCS stimulation, mostly after cathodal stimulation. Nonetheless no differences between groups were found according to Mann-Whitney U-test for independent samples (*cf.* S1 Table).

**Psychological questionnaires**

## In day 1, independent samples *t*-tests for each psychological measure showed no baseline differences between groups. Similarly, state anxiety (STAI 1) was not different between groups in day 2 and 3, suggesting that tDCS stimulation did not impact self-reported symptoms (*cf.* S2 Table).

**References**

1. Boucsein W. Electrodermal Activity. Boucsein W, editor. Springer US; 2012.
2. Lebron-Milad K, Milad MR. Sex differences, gonadal hormones and the fear extinction network: implications for anxiety disorders. Biol Mood Anxiety Disord [Internet]. 2012 Feb 7;2:3. Available from:  [https://www.ncbi.nlm.nih.gov/pubmed/22738383](../%20https://www.ncbi.nlm.nih.gov/pubmed/22738383)
3. Boddez, Y., Baeyens, F., Luyten, L., Vansteenwegen, D., Hermans, D., & Beckers, T. (2013). Rating data are underrated: Validity of US expectancy in human fear conditioning. Journal of Behavior Therapy and Experimental Psychiatry, 44(2), 201–206. Available from: <https://doi.org/https://doi.org/10.1016/j.jbtep.2012.08.003>
4. Lonsdorf TB, Menz MM, Andreatta M, Fullana MA, Golkar A, Haaker J, et al. Don’t fear ‘fear conditioning’: Methodological considerations for the design and analysis of studies on human fear acquisition, extinction, and return of fear. Neurosci Biobehav Rev [Internet]. 2017;77:247–85. Available from: <http://www.sciencedirect.com/science/article/pii/S0149763416308466>
5. Raij T, Nummenmaa A, Marin M-F, Porter D, Furtak S, Setsompop K, et al. Prefrontal Cortex Stimulation Enhances Fear Extinction Memory in Humans. Biol Psychiatry [Internet]. 2018 Jul 15;84(2):129–37. Available from: <https://doi.org/10.1016/j.biopsych.2017.10.022>
6. Golkar A, Tjaden C, Kindt M. Vicarious extinction learning during reconsolidation neutralizes fear memory. Behav Res Ther [Internet]. 2017;92:87–93. Available from: <http://www.sciencedirect.com/science/article/pii/S0005796717300396>
7. Boucsein W, Fowles DC, Grimnes S, Ben-Shakhar G. Publication recommendations for electrodermal measurements. Psychophysiology [Internet]. 2012 Aug 1;49(8):1017–34. Available from: <https://doi.org/10.1111/j.1469-8986.2012.01384.x>
8. Krypotos A-M, Effting M, Kindt M, Beckers T. Avoidance learning: a review of theoretical models and recent developments. Front Behav Neurosci [Internet]. 2015 Jul 21;9:189. Available from: <https://www.ncbi.nlm.nih.gov/pubmed/26257618>
